# Current Development of iPSC-Based Modeling in Neurodegenerative Diseases

**DOI:** 10.3390/ijms26083774

**Published:** 2025-04-16

**Authors:** Xiangge Guo, Xumeng Wang, Jiaxuan Wang, Min Ma, Qian Ren

**Affiliations:** 1 Department of Human Anatomy, Hebei Medical University, Shijiazhuang 050017, China; ge13710230621@163.com (X.G.); wangxm001103@163.com (X.W.); 18333323195@163.com (J.W.); 2 Human Brain Bank, Hebei Medical University, Shijiazhuang 050017, China; 3 The Key Laboratory of Neural and Vascular Biology, Ministry of Education, Hebei Medical University, Shijiazhuang 050017, China; 4 Hebei Key Laboratory of Neurodegenerative Disease Mechanism, Hebei Medical University, Shijiazhuang 050017, China

**Keywords:** neurodegenerative disease, iPS cell, brain organoid, reprogramming factor, neural stem cell, neuron, microglia, astrocyte, oligodendrocyte

## Abstract

Over the past two decades, significant advancements have been made in the induced pluripotent stem cell (iPSC) technology. These developments have enabled the broader application of iPSCs in neuroscience, improved our understanding of disease pathogenesis, and advanced the investigation of therapeutic targets and methods. Specifically, optimizations in reprogramming protocols, coupled with improved neuronal differentiation and maturation techniques, have greatly facilitated the generation of iPSC-derived neural cells. The integration of the cerebral organoid technology and CRISPR/Cas9 genome editing has further propelled the application of iPSCs in neurodegenerative diseases to a new stage. Patient-derived or CRISPR-edited cerebral neurons and organoids now serve as ideal disease models, contributing to our understanding of disease pathophysiology and identifying novel therapeutic targets and candidates. In this review, we examine the development of iPSC-based models in neurodegenerative diseases, including Alzheimer’s disease, Parkinson’s disease, and Huntington’s disease.

## 1. Introduction

Since the advent of induced pluripotent stem cells (iPSCs) in 2006, two decades of advancements have enabled the widespread application of iPSCs, their differentiated cells and organoids in modeling neurological diseases, elucidating pathogenic mechanisms, and advancing cell-based therapies [1]. Previous reviews have extensively discussed the advantages of iPSC-derived patient-specific in vitro cellular models over traditional animal models and human embryonic stem cells, particularly in disease-specific applications [1,2,3]. However, significant limitations persist in recapitulating disease-specific pathological phenotypes and mechanistic pathways using iPSC-derived cells and organoids [3,4,5]. For instance, heterogeneity and dosage variability remain critical challenges due to differences in cellular/tissue sources, induction protocols, and culture conditions [6]. Furthermore, the existing induction protocols still face technical complexities and cannot indefinitely generate all desired human cell types [7,8].

To address these challenges, researchers have continuously improved iPSC generation methods and differentiation protocols. Simultaneously, the expanding application of the CRISPR/Cas9 gene-editing technology has enabled precise genetic modifications in iPSCs, their differentiated cells, and even organoids, while preserving patient-specific genetic backgrounds [9,10]. These advances have elevated research on disease-specific model establishment, mechanistic investigation, and high-throughput drug screening to new heights. This review systematically elaborates on the recent progress in iPSC induction and iPSC differentiation into neurons, glial cells, and organoids, while examining the applications of these cellular and organoid systems as in vitro disease models for neurodegenerative disorders.

## 2. Generation of iPSCs

### 2.1. Delivery Methods

The initial generation of iPSCs relied on the transient expression of exogenous transcription factors [2]. Conventional delivery methods involve retroviral and lentiviral vectors to deliver transcription factors in somatic cells [11]. While these retroviral and lentiviral systems are highly efficient and robust, they carry a significant risk of transgene reactivation post-reprogramming [12]. With advancements in gene delivery technologies, a diverse array of reprogramming methods has been developed. Currently, the most commonly employed strategies include non-integrating viral approaches—such as adenovirus, Sendai virus, and protein delivery—and non-viral methods, including mRNA transfection, PiggyBac transposons, minicircle vectors, and episomal plasmids [2,11,13]. Each of these approaches has its own set of advantages and limitations (Table 1). For example, adenovirus vectors, while associated with a lower risk of transgene reactivation, exhibit suboptimal reprogramming efficiency, making them unsuitable for clinical applications [11,13]. In contrast, Sendai virus-based reprogramming is considered safer and more efficient, as the RNA virus can be eliminated from iPSCs, reducing the risk of genomic integration [11,13]. DNA-based reprogramming methods, such as episomal plasmids, PiggyBac transposons, and minicircle vectors, offer distinct benefits. PiggyBac and minicircle vectors are associated with reduced risks of genomic instability and mutations. However, these methods are limited by low reprogramming efficiency and the restricted availability of transducible somatic cell types [14,15,16]. Episomal plasmids, containing EBNA-1 and OriP sequences from Epstein–Barr virus, represent an alternative DNA-based method. While daily transfection is required to enhance reprogramming efficiency, episomal plasmids circumvent the risk of genomic integration. Moreover, they are cost-effective, easier to produce, and more straightforward to use compared to PiggyBac and minicircle vectors [17,18]. In addition to DNA-based approaches, RNA delivery has emerged as another viable approach to induce pluripotency [19]. This method demonstrates a lower mutagenic risk and a higher efficiency; however, its application has so far been limited to reprogramming human fibroblasts and peripheral blood cells [20,21].

### 2.2. Reprogramming Factors

Takahashi and Yamanaka initially established iPSCs by overexpressing the four transcription factors, OCT3/4, SOX2, KLF4, and c-MYC (OSKM), in mouse fibroblasts [22]. The four factors, often referred to as Yamanaka factors, perform distinct yet complementary roles: OCT3/4, SOX2, and KLF4 are essential for maintaining pluripotency and inhibiting differentiation, while c-MYC enhances reprogramming efficiency and promotes cell proliferation [23]. In addition to OSKM, various other combinations of transcription factors have been identified to induce iPSC reprogramming (Table 2). For example, OCT3/4, SOX2, NANOG, and LIN28 represent an alternative set of transcription factors, where NANOG plays a pivotal role in stem cell self-renewal, and LIN28 regulates RNA modification and expression [24]. Additionally, transcription factors such as GLIS1, NR5A2, and SALL4 can substitute for c-MYC or OCT3/4, or complement OSKM to enhance the reprogramming efficiency and improve the pluripotent state of the cells [25,26,27].

Recent studies have further demonstrated that epigenome modifiers and miRNA manipulation can significantly enhance iPSC reprogramming efficiency [2] (Table 2). Chemical approaches have also been developed to regulate the pluripotency and differentiation of iPSCs. These methods typically target growth factor receptors or downstream kinases that modulate intracellular signaling pathways during differentiation. In 2011, Yu et al. reported the CHALP cocktail, a combination of six small molecules: CHIR99021 (a GSK3β inhibitor), PD0325901 (a MEK inhibitor), human leukemia inhibitory factor (LIF), A-83-01 (a TGF-β/activin/nodal receptor inhibitor), basic fibroblast growth factor (bFGF), and HA-100 (a ROCK inhibitor), which collectively enhance reprogramming efficiency [28]. Another chemical cocktail protocol includes cyclic pifithrin-α (a P53 inhibitor), A-83-01, CHIR99021, thiazovivin, sodium butyrate (NaB), and PD0325901, which has shown particular efficacy in reprogramming human urine-derived cells (hUCs) [29]. These cocktails exert complex biological effects, such as promoting ground-state pluripotency and facilitating iPSC production from neural progenitor cells [30].

Additionally, specific chemicals, including DNA methyltransferase inhibitors (such as 5′azacytidine (AZA) or valproic acid (VPA)) and histone deacetylase inhibitors (such as trichostatin A, suberoylanilide hydroxamic acid), have been shown to enhance reprogramming efficiency [30,31,32] (Table 2). For example, AZA or VPA can achieve reprogramming without the introduction of the oncogenes c-Myc and Klf4, and the efficiency is significantly increased. Trichostatin A and suberoylanilide hydroxamic acid can improve reprogramming efficiency by more than two-fold under OSKM conditions. Furthermore, reports have shown that the small molecule combination BIX-01294 (a G9a histone methyltransferase inhibitor) and BayK8644 (an L-type calcium channel agonist) enable reprogramming of Oct4/Klf4-transduced fibroblasts. These chemical approaches hold significant promise for advancing stem cell research and accelerating applications in regenerative medicine.

### 2.3. Somatic Cell Sources

Due to the accessibility and rapid proliferation of fibroblasts, iPSCs were initially successfully induced from mouse fibroblasts. Currently, in addition to human and mouse cells, fibroblasts from various mammalian species, including pigs, rabbits, monkeys, and horses, have been successfully reprogrammed into iPSCs [33,34,35,36,37]. Among human somatic cells, skin fibroblasts remain a primary source for iPSCs [38]. However, the invasive nature of skin biopsies often limits their practicality and patient acceptance.

With advancements in reprogramming technologies, iPSCs can now be derived from various somatic cell types beyond skin fibroblasts [38,39,40]. Peripheral blood mononuclear cells (PBMCs), including T cells, B cells, and monocytes, are particularly valuable due to their accessibility through non-invasive collection methods, making them a promising resource for the generation of iPSC, especially for clinical applications [41,42,43,44,45,46,47]. Keratinocytes, isolated from skin or hair, also provide a non-invasive option for iPSC generation [48]. Mesenchymal stem cells (MSCs), derived from tissues such as bone marrow, adipose tissue, and teeth, are another accessible and widely utilized source, particularly in studies involving regeneration and differentiation [38,49]. Renal epithelial cells isolated from urine represent one of the most convenient and non-invasive sources for iPSC induction [39,50]. Neural stem cells (NSCs) and neural progenitor cells (NPCs), given their inherent pluripotency, have also been successfully reprogrammed into iPSCs, facilitating research in neurological disease modeling [51]. Additionally, cells from other sources, such as liver, stomach, and cord blood, have demonstrated viability for iPSC induction, further expanding the applications of iPSCs in both research and therapeutic contexts [52,53].

Although nearly all somatic cells exhibit the potential for reprogramming into iPSCs, their accessibility and reprogramming efficiency vary significantly. Therefore, careful selection of an appropriate cell source, reprogramming factors, and delivery method is critical for the successful establishment of iPSCs, particularly for disease modeling and subsequent applications (Table 3). Notably, iPSCs retain the epigenetic memory of their original somatic cell type, which can influence both the reprogramming efficiency and the differentiation potential [54]. This retained epigenetic status plays a pivotal role in determining the functional capacity of iPSCs and must be carefully considered in both research and clinical settings.

## 3. Neural Differentiation of iPSCs

In the pathogenesis of neurodegenerative diseases, neurons, glial cells, and other cell types play pivotal roles. However, obtaining these cells has long posed a significant challenge for researchers. The advent of iPSCs has greatly alleviated the difficulties associated with accessing these cell types. Currently, various neural cells derived from iPSCs have been widely utilized in numerous fields of neuroscience. Particularly in the application as in vitro disease models, iPSC-derived neural cells have demonstrated broad potential.

**Figure 1:**
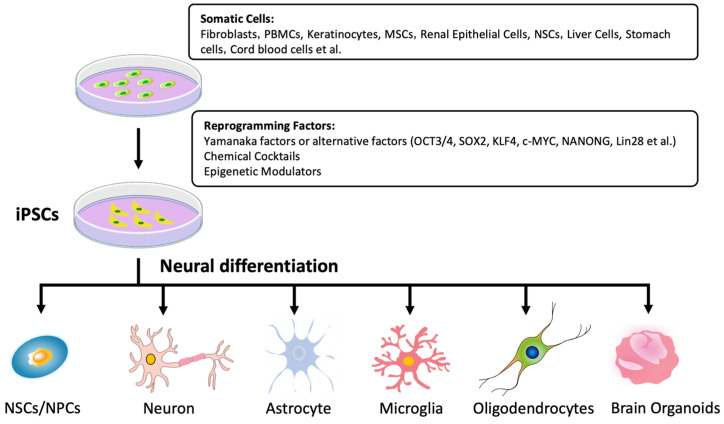
In addition to fibroblasts, PBMCs, and NSCs, a wide range of somatic cell sources can be reprogrammed in vitro using methods such as OSKM or chemical cocktails to establish iPSCs. These iPSCs can then be further differentiated into various neurons and organoids, which are widely utilized as disease models in the study of neurodegenerative disorders.

Obtaining neurons and other relevant cells from patients with neurodegenerative diseases has traditionally posed significant challenges, making it difficult to replicate pathological processes in vitro. However, iPSCs provide a promising solution due to their ease of acquisition, extensive proliferative and differentiation capacities, and minimal ethical concerns [39]. Furthermore, iPSCs retain the genetic background of donor tissues and organs, enabling them to accurately model human physiological and pathological characteristics in vitro [55]. As a result, iPSC-derived neurons have become widely utilized for investigating the underlying mechanisms of neurodegenerative diseases and for screening potential therapeutic targets and drugs (Figure 1 and Table 4).

### 3.1. Neural Stem Cells

Neural differentiation from iPSCs typically involves inducing iPSCs into NPCs or NSCs using one of three main strategies: embryoid body (EB) formation, co-culture on neural-inducing feeder layers, or dual SMAD inhibition [56,57]. These derived NSCs/NPCs can be expanded in adherent cultures or as floating neurospheres and subsequently treated with specific growth factors to drive differentiation into various neuronal subtypes [57]. Additionally, some direct induction methods bypass the NSC/NPC stage, enabling direct neural induction [3]. Regardless of the approach, iPSC-derived neurons offer unprecedented opportunities for the development of disease models and high-throughput drug screening.

### 3.2. Neurons

As described above, most neuronal cell types can be induced through the neural rosettes and NSC/NPC stages, followed by the addition of specific growth factors or small molecules to further guide their differentiation into specialized neuronal subtypes. For example, supplementation with brain-derived neurotrophic factor (BDNF), glial cell line-derived neurotrophic factor (GDNF), cyclic adenosine monophosphate (cAMP), forskolin, or retinoic acid (RA), as well as activators of WNT and Sonic Hedgehog (SHH) signaling pathways, can promote the generation of diverse neuronal types, including dopaminergic neurons, GABAergic neurons, and glutamatergic neurons [3,58,59,60,61,62,63].

In addition to protocols that recapitulate developmental differentiation processes in vitro, functional induced neurons (iNs) can also be obtained through direct conversion methods from iPSCs, or even directly from somatic cells [64,65,66,67,68,69,70,71,72,73,74]. These methods significantly shorten the differentiation time, lower costs, and simplify procedures [71]. For example, direct conversion of iPSCs to iNs can be achieved by overexpressing specific neurodevelopmental transcription factors such as BRN2, ASCL1, and MYT1L or NGN2 [67]. Furthermore, overexpression of ASCL1 and DLX2 in iPSCs has been shown to efficiently generate GABAergic neurons [68,69]. Similarly, a combination of SMAD and SHH inhibition with the overexpression of transcription factor NGN2 can directly induce the formation of mature glutamatergic neurons [70]. Additionally, neurons can also be obtained directly from somatic cells through trans-differentiation. For instance, fibroblasts can be reprogrammed into iNs by overexpressing BRN2, ASCL1, MYT1L, and NEUROD1 [73]. Other studies have demonstrated that combinations of certain microRNAs (e.g., miR-9/9*, miR-124) and transcription factors (e.g., MYT1L, NEUROD2) can convert fibroblasts into iNs [64,65,66,74].

### 3.3. Astrocytes

Astrocytes are the most abundant glial cells in the human brain [75]. Several protocols have been established to differentiate iPSCs into astrocytes [57,75,76,77]. The predominant strategies involve differentiating iPSCs through the NSC/NPC or oligodendrocyte progenitor cell (OPC) stages, followed by further maturation into astrocytes using a combination of growth factors and small molecules. For instance, the differentiation of NSCs/NPCs into astrocytes can be accelerated by using a combination of ciliary neurotrophic factor (CNTF), bone morphogenetic proteins (BMP), FGF2, and fetal bovine serum (FBS) [76,78,79]. Similarly, a mixture of N2, B27-RA, BMP4, and FGF2 has also been reported to facilitate the differentiation of OPCs or NPCs into astrocytes [80]. Beyond strategies that undergo physiological developmental stages, direct generation of astrocytes from iPSCs has been demonstrated by inducing the expression of transcription factors such as NFIA or NFIA in combination with SOX10 [79,80,81,82]. Additionally, direct conversion of fibroblasts into astrocytes has been achieved through the overexpression of NFIA, NFIB, and SOX9 [72]. However, it is important to note that astrocytes exhibit significant plasticity, which limits the ability of in vitro-cultured astrocytes to fully replicate their in vivo counterparts. Moreover, variability between cell lines derived using different differentiation protocols remains a notable challenge that warrants further investigation [83,84].

### 3.4. Microglia

Microglia, as the innate immune cells of the central nervous system (CNS), play a critical role in neural development, homeostasis, and repair [85]. Similar to iPSC-derived neurons and astrocytes, microglia can be differentiated from iPSCs using various established protocols. The most widely used approach involves initially inducing iPSCs into mesoderm progenitor cells, followed by directed differentiation into microglia. These approaches typically begin with the addition of specific cytokines such as BMP4, activin A, FGF2, and vascular endothelial growth factor A (VEGF-A) to promote the formation of yolk sac embryoid bodies (EBs) or hematopoietic progenitor cells (HPCs). Subsequently, cells are exposed to factors such as interleukin 34 (IL-34), TGF-β, and cell survival factors (CSF) to generate microglia-like cells [85,86,87,88,89]. These protocols aim to recapitulate the in vivo developmental process of microglia. However, they have certain limitations, including multiple steps, high technical complexity, and relatively low differentiation efficiency [89]. Alternative methods have been developed to directly differentiate iPSCs into microglia by introducing key transcription factors such as PU.1 and interferon regulatory factor 8 (IRF8) [90,91]. Additionally, co-culture systems with astrocytes or neurons can provide essential microenvironmental factors to facilitate further differentiation into microglia-like cells [89,92]. It is important to note that microglia-like cells generated in vitro often exhibit phenotypic and functional differences from their in vivo counterparts. Variations in functional characteristics such as phagocytic activity and inflammatory responses are observed across different differentiation protocols [85,87,89]. Nevertheless, mesoderm-derived microglia tend to closely resemble primary microglia and are considered more suitable for modeling neurodegenerative diseases and investigating immune responses within the CNS [89].

### 3.5. Oligodendrocytes

The primary function of oligodendrocytes is to produce myelin, which protects neurons and axons while maintaining their connectivity. Demyelination of neurons is a common pathological feature observed in neurodegenerative diseases [93]. Consequently, oligodendrocytes derived from iPSCs hold significant potential for studying neurodegenerative disorders. The differentiation of oligodendrocytes typically follows a stepwise process: iPSCs are first induced into NPCs, then differentiated into oligodendrocyte progenitor cells (OPCs), and finally matured into functional oligodendrocytes [94]. During this process, growth factors such as BMP4, FGF2, EGF, and PDGF are used to promote the transition from NPCs to OPCs [94,95]. The subsequent addition of myelinogenic factors, including triiodothyronine (T3) and insulin-like growth factor 1 (IGF-1), facilitates the maturation of OPCs into oligodendrocytes [96,97]. These methods demonstrate a relatively high induction efficiency and yield oligodendrocytes with stable functionality. However, the protocols are complex, time-consuming, and technically challenging.

Alternatively, the direct overexpression of transcription factors such as SOX10, OLIG2, and NKX6.2 in iPSCs has been shown to expedite differentiation into OPCs [98,99]. This approach significantly shortens the differentiation timeline, but the introduction of transcription factors may cause phenotypic instability [94]. Furthermore, the application of small molecules, such as SMAD inhibitors and ROCK inhibitors, combined with such factors as RA, EGF, FGF2, and SHH, has also been shown to drive NPCs toward oligodendrocyte differentiation [94,97]. Recent studies have reported that combining IGF-1 with promyelinating agents such as clemastine and ketoconazole enhances the differentiation of OPCs into mature oligodendrocytes [100,101]. Additionally, co-culture systems involving OPCs with mature neurons and astrocytes can provide essential microenvironmental factors, such as CX3CL1 and TGF-β, which are required during the differentiation process. These systems promote the generation of mature oligodendrocytes with enhanced myelination capacity [102,103]. Although these strategies are effective in producing oligodendrocytes with higher maturation levels, challenges remain in achieving high cell purity.

### 3.6. Brain Organoids

Although iPSC-derived cells effectively model various functional and structural abnormalities at the cellular level in vitro, these culture systems are limited in their ability to replicate the complex features of cell–cell interactions and microenvironmental cues present in vivo [104]. Brain organoids, three-dimensional (3D) structures that spontaneously self-organize into adequately differentiated cell types, have emerged as powerful tools for simulating human brain development in vitro [105,106,107]. Compared to traditional two-dimensional (2D) cell culture systems, 3D brain organoids better mimic the in vivo environment and are thus more suitable for modeling neurobiological processes of the human brain. As a result, brain organoids have become indispensable tools for investigating the pathogenesis of neurodegenerative diseases and for performing high-throughput drug screening [108,109,110].

Current protocols for generating brain organoids from iPSCs can be broadly categorized into non-guided and guided differentiation methods [108,111,112,113,114]. The non-guided differentiation method relies on the cells’ intrinsic morphogenetic potential rather than on exogenous inductive factors. This method begins with the suspension culture of iPSCs to form EBs, which then develop into neuroectodermal-like structures. These structures are encapsulated in Matrigel and maintained in a 3D culture system using a bioreactor, allowing them to gradually differentiate into brain organoids composed of diverse cell types [105,115]. Brain organoids generated through this method typically exhibit multiple brain-like regions (e.g., forebrain, midbrain) and high cellular diversity, enabling the simulation of developmental characteristics across different brain regions. However, due to the absence of exogenous inducers, this method lacks precise control over cell differentiation and brain region specification, resulting in poor repeatability [105,115,116].

In contrast, the guided differentiation method employs exogenous factors to direct cell fate. After forming EBs in a standard suspension culture, a dual SMAD inhibitor is added to promote neural lineage differentiation. Subsequently, region-specific morphogenic and neurotrophic factors are introduced, and the cells are transferred to a Matrigel-encapsulated 3D culture system for long-term cultivation, enabling the formation of region-specific organoids, such as cortical, hippocampal, or midbrain organoids [108,111,112,113,114,116]. For example, forebrain neural precursor cells can be generated by inhibiting the WNT signaling pathway using IWR-1 and retinoic acid (RA), combined with FGF2 and EGF to maintain proliferation and drive differentiation into cortical organoids [117,118]. Similarly, adding SHH and FGF8 during the neuroectodermal stage can induce midbrain progenitor cells, while the addition of BDNF, GDNF, ascorbic acid, and cAMP promotes the formation of midbrain organoids containing functional dopaminergic neurons [119,120,121]. Hippocampal organoids can be generated by inhibiting WNT, TGF-β, SHH, and BMP signaling pathways to produce hippocampus-patterned NPCs (hpNPCs), followed by exposure to WNT3A, BDNF, cAMP, and ascorbic acid, yielding hippocampal organoids containing VGLUT1+, NeuN+, and glutamatergic neurons [122,123,124].

Additionally, other protocols have been established to generate various specialized brain organoids, including cerebellar organoids, retinal organoids, and choroid plexus organoids, further expanding the applications of 3D brain models in neurobiological research [104,106,109,125]. Notably, despite significant advancements, brain organoids still face challenges in fully recapitulating the physiological features of the human brain, primarily due to the lack of vascularization and cellular heterogeneity [109,110].

### 3.7. Assembloids

Assembloids are 3D models formed by integrating multiple organoids or cell types, allowing the study of complex cellular interactions in disease models, including neurodegenerative disorders. These models enable the simulation of neural migration, axon guidance, and circuit formation, mimicking both inter-regional and intra-regional cell–cell interactions within the nervous system. Assembloids have been particularly useful in studying interactions between neural and non-neural cells, such as vascular and immune cells, thereby enhancing the physiological relevance of in vitro systems. The integration of multiple organoid types into assembloids has advanced understanding of developmental processes and disease mechanisms, offering a promising tool for disease modeling, including those for neurodegenerative diseases [126].

## 4. Neurodegenerative Disease Modeling with Patient-Derived iPSCs

Induced pluripotent stem cells (iPSCs) and their derivatives possess an identical genome to that of the donor patient, making patient-derived iPSCs invaluable as in vitro models for studying neurodegenerative diseases that previously lacked appropriate models. While iPSCs have the potential to investigate any neurodegenerative disease, this review specifically focuses on three prevalent conditions: Alzheimer’s disease (AD), Parkinson’s disease (PD), and Huntington’s disease (HD). Although the majority of these cases are sporadic, iPSC-derived cells from patient iPSCs often exhibit phenotypes consistent with familial cases, despite differences in the underlying molecular mechanisms [2]. The establishment of such models is critical for elucidating the precise pathogenesis of these diseases and for the development of targeted therapeutic strategies for both sporadic and familial forms.

However, several limitations must be considered when applying the iPSC technology to study these diseases. A key challenge is the variability in the maturity and functionality of the differentiated somatic cells, which can lead to inconsistencies in experimental observations. This variability is observed even in iPSCs derived from the same individual, complicating the accurate assessment of disease phenotypes. Such differences may arise from genetic variations between clones, epigenetic modifications, the source of iPSCs, or the presence of residual transgenes in each iPSC clone [54,127]. Addressing these issues is crucial to improving the quality of iPSC-based disease models and increasing their reliability for research purposes.

### 4.1. Alzheimer’s Disease

In the case of AD, the majority of cases are sporadic AD (sAD), accounting for approximately 95%, while familial AD (fAD) represents only about 5%, where fAD is typically associated with mutations in the amyloid precursor protein (APP), PSEN1, and PSEN2 genes. In contrast, the APOE4 allele is a primary risk factor in sAD, with approximately 65–80% of sAD patients carrying this allele, while the APOE2 allele is considered protective. Additionally, over 20 risk genes associated with AD, such as SORL1 and TREM2, have been identified. A key pathological hallmark of AD is the deposition of β-amyloid (Aβ) peptides. The formation of Aβ plaques (primarily Aβ42) involves the sequential cleavage of APP by β- and γ-secretase, while α-secretase cleavage of APP has a protective effect. PSEN1 and PSEN2 constitute the catalytic core of γ-secretase. Another significant pathological characteristic of AD is the aggregation of the Tau protein in neurons, leading to the formation of neurofibrillary tangles. Hyperphosphorylation of Tau, driven by kinases such as glycogen synthase kinase 3β (GSK-3β) and cyclin-dependent kinase 5 (CDK5), results in neuronal tangles. However, the specific mechanisms underlying these pathological features, as well as the interplay between phosphorylated Tau (p-Tau) and Aβ, remain unclear. Consequently, iPSCs carrying these genetic mutations offer powerful tools for investigating these mechanisms [128,129,130].

Most researchers focus on neurons derived from iPSCs of AD patients, particularly those with mutations in APP, PSEN1, PSEN2, and APOE4, associated with either fAD or sAD. AD neurons derived from iPSCs replicate key pathological features of AD, including Aβ accumulation and Tau hyperphosphorylation [131,132,133]. These neurons also exhibit pathological phenotypes such as activation of GSK3β, abnormal neuronal electrophysiological activity, increased oxidative stress, elevated reactive oxygen species (ROS) production, endoplasmic reticulum dysfunction, and mitochondrial abnormalities [134,135,136,137,138]. AD neurons show a heightened sensitivity to Aβ42 compared to healthy individuals, and treatment with β- or γ-secretase inhibitors has been shown to downregulate Aβ secretion and p-Tau levels [128,131,133,135,139,140]. These findings establish neurons derived from AD patients as robust in vitro models for investigating AD mechanisms and drug discovery (Table 5).

In addition to neurons, researchers have utilized other iPSC-derived in vitro models for AD, including NSCs/NPCs, microglia, astrocytes, and oligodendrocytes. Studies on AD-NSCs reveal that NSCs from fAD or sAD patients with APOE and PSEN1 mutations express low levels of APP and Aβ without notable morphological differences. However, their proliferation and self-renewal capabilities are significantly impaired, and they exhibit elevated expression of neurodevelopmental genes such as MAPT, CD24, and STMN2 [131,141,142]. These findings suggest that while AD-NSCs may not display the AD pathology, their compromised proliferation and differentiation functions indicate that AD-NSCs may serve as a valuable in vitro model for studying early-stage AD and environmental risk factors (Table 5).

AD-derived microglia display diminished phagocytic capacity for Aβ and Tau oligomers, alongside increased neuroinflammation [85,143,144]. Astrocytes derived from fAD (PSEN1) and sAD (APOE3/4) patients exhibit morphological alterations, increased Aβ42 release, impaired clearance capabilities, dysregulated cytokine production, calcium homeostasis imbalance, and elevated ROS levels [145,146,147,148]. Although relatively few studies have focused on AD iPSC-derived oligodendrocytes, evidence suggests they suffer from morphological, proliferative, and functional impairments, impacting their neuronal support and myelin formation capabilities [149] (Table 5).

To investigate the interactions between cell types and the formation of pathological features in complex tissue environments, 3D brain organoids derived from iPSCs have gained significant attention. Studies have reported that introducing pathogenic mutations in APP or PSEN1 into NPCs and differentiating them into brain organoids can reveal Aβ deposition and neurofibrillary tangle formation [150]. Brain organoids derived from fAD and sAD iPSCs show increased Aβ production, enhanced Tau phosphorylation, and endoplasmic reticulum abnormalities [151,152,153,154,155]. Furthermore, these pathological changes are modifiable through pharmacological and environmental interventions. Thus, AD brain organoids are highly valuable for studying cell–cell interactions, the mechanisms underlying pathological structure formation, and the effects of therapeutic strategies in a 3D context (Table 5).

Numerous studies have focused on establishing iPSC-based Alzheimer’s disease (AD) models using the CRISPR gene editing technology. For instance, CRISPR/Cas9-generated astrocytes carrying APP or PSEN1 mutations exhibit increased Aβ production, oxidative stress, and impaired neuronal function [145,156]. Neurons engineered to express APOE4 via gene editing demonstrate a significantly elevated Aβ production, heightened tau phosphorylation, and degeneration of GABAergic neurons [157]. Furthermore, microglia with CRISPR/Cas9-introduced TREM2 R47H mutations show reduced lipid droplet accumulation, altered plaque reactivity, and modified APOE secretion patterns [158].

### 4.2. Parkinson’s Disease

Parkinson disease (PD) is the second most common neurodegenerative disease after Alzheimer’s disease. Approximately 10% of PD patients are diagnosed with familial Parkinson’s disease (fPD), while the remaining cases are classified as sporadic Parkinson’s disease (sPD) [159]. The primary pathological hallmark of PD is the progressive loss of dopaminergic (DA) neurons, accompanied by the accumulation of α-synuclein and the formation of Lewy bodies [160]. Mutations in genes such as SNCA, PARK2, PINK1, and LRRK2 are closely associated with the onset of both fPD and sPD, although the precise roles of these mutations in PD pathogenesis remain unclear [2,161]. Therefore, the use of patient-derived DA neurons with these genetic mutations offers a powerful platform for elucidating the mechanisms underlying PD and screening potential therapeutic agents.

In fPD, DA neurons derived from PD-iPSCs carrying mutations in SNCA, LRRK2, PARK2, PINK1, and the β-glucocerebrosidase gene (GBA1) exhibit characteristic pathological features of PD [2,5,159,162]. SNCA encodes α-synuclein, and studies have consistently shown that DA neurons derived from patients with SNCA triplications or gene multiplication exhibit elevated α-synuclein protein levels, increased sensitivity to oxidative and endoplasmic reticulum (ER) stress, mitochondrial dysfunction, synaptic loss, and increased neuronal death [163,164,165,166,167,168,169]. Similarly, DA neurons harboring the SNCA–A53T mutation display abnormal α-synuclein aggregation, mitochondrial dysfunction, and increased apoptosis [170,171]. Mutations in the LRRK2 gene are another common cause of fPD [172]. Studies have reported that LRRK2-mutant DA neurons exhibit elevated SNCA transcription, mitochondrial dysfunction, oxidative stress, increased apoptosis, and impaired neuronal homeostasis [173,174,175,176,177,178,179] (Table 6).

Although PD primarily affects individuals over 65 years of age, approximately 5–10% of cases are classified as early-onset PD (EOPD) [180]. Mutations in PARK2 and PINK1 are strongly associated with EOPD [181]. DA neurons derived from PARK2 and PINK1 iPSCs exhibit typical PD-like pathological features, including α-synuclein accumulation, defects in mitochondrial autophagy, elevated ROS levels, and increased sensitivity to oxidative stress [182,183,184,185]. These pathological features can be ameliorated using pharmacological agents such as coenzyme Q10 and rapamycin [186]. While sPD accounts for the majority of PD cases, studies have also identified α-synuclein accumulation and epigenetic changes in sPD-derived DA neurons, often associated with single-gene mutations such as LRRK2, although such studies remain limited. Additionally, rarer forms of PD, such as those caused by GBA1 and VPS35 mutations, exhibit similar pathological phenotypes, including α-synuclein accumulation, mitochondrial damage, and elevated ROS production in DA neurons [105,187,188]. These iPSC-derived cells provide invaluable in vitro models for investigating PD pathogenesis and screening potential therapies (Table 6).

Beyond DA neurons, iPSC-derived astrocytes, microglia, and neural stem cells have also garnered significant attention. For instance, astrocytes derived from LRRK2-mutant iPSCs exhibit α-synuclein accumulation, dysregulated autophagy, abnormal mitochondrial morphology and activity, and reduced viability, leading to oxidative stress and degeneration of DA neurons [189,190,191]. Similarly, SNCA- and LRRK2-mutant iPSC-derived microglia display elevated α-synuclein levels, impaired phagocytic capacity, and increased inflammation, ultimately affecting DA neuron function and morphology [191,192,193]. Furthermore, neural stem cells derived from LRRK2-mutant iPSCs exhibit reduced differentiation efficiency and developmental defects [194,195] (Table 6).

Most current in vitro models of PD primarily rely on 2D cell cultures, which lack the complex cellular interactions and multilayered structural features necessary for accurately modeling disease pathology and evaluating drug efficacy. To address these limitations, 3D brain organoids have emerged as a promising tool in PD research [104]. For example, LRRK2-mutant DA neurons cultured in a 3D environment exhibited increased cell death, reduced differentiation potential, and decreased dendritic complexity compared to controls, and these phenotypes can be partially rescued by inhibiting LRRK2 [196]. Similarly, studies have demonstrated a significant reduction in DA neuron populations and neurodevelopmental defects in LRRK2-mutant iPSC-derived organoids [197]. These findings highlight 3D organoids as advanced in vitro models for elucidating PD pathogenesis and developing novel therapeutic strategies (Table 6).

CRISPR-edited iPSC models have been instrumental in studying PD pathogenesis, with key modifications targeting several disease-associated genes. SNCA models through intron-4 enhancer SNP editing or triplication correction demonstrate reduced α-synuclein aggregation and improved neuronal differentiation [163,198]. LRRK2 G2019S mutation correction restores neurite length and reduces phospho-α-synuclein levels while normalizing the astrocyte lysosomal function [189,199]. GBA N370S mutation correction, achieved through precise editing that avoids the GBAP1 pseudogene, improves glucocerebrosidase activity and reduces α-synuclein accumulation [200,201]. PINK1/PRKN single and double knockout models reveal mitophagy dysfunction, increased oxidative stress, and PRKN-specific lysosomal impairment [202,203]. Additionally, CHCHD2/CHCHD10 knockout models show compromised mitochondrial respiration and motor neuron vulnerability [204]. These models collectively highlight the importance of mitochondrial/lysosomal pathways in PD and demonstrate how 3D systems better recapitulate pathology than traditional 2D cultures. The development of these genetically precise iPSC models has significantly advanced our understanding of PD mechanisms and provided valuable platforms for therapeutic development.

### 4.3. Huntington’s Disease

Huntington’s disease (HD) is an autosomal dominant hereditary neurodegenerative disorder characterized by progressive motor dysfunction, cognitive decline, and psychiatric disturbances. HD is caused by an abnormal expansion of the CAG trinucleotide repeat within exon 1 of the huntington gene (HTT) located on chromosome 4. The length of CAG repeats is inversely correlated with the age of disease onset and directly correlated with disease severity. Pathologically, HD is defined by hallmark features including the aggregation of mutant HTT, progressive striatal degeneration with the selective loss of medium spiny neurons (MSNs), disruptions in neurotransmitter homeostasis, mitochondrial dysfunction, and prominent extrapyramidal motor abnormalities, such as chorea and dystonia [205].

Recent advances in the generation of in vitro models from HD-iPSCs have provided significant insights into HD pathogenesis (Table 7). Notably, the HD Consortium successfully differentiated 14 HD-iPSC lines, encompassing both early-onset and late-onset cases, into NPCs/NSCs, forebrain neurons, and striatal-like neurons. These HD-derived cells express mutated HTT with expanded CAG repeats, and transcriptomic analyses reveal dysregulation in genes associated with key pathways, including signal transduction, cell cycle, axonal guidance, neuronal development, cytoskeleton organization, cell adhesion, and cellular metabolism. These molecular changes are consistent with the known transcriptional abnormalities in HD and are accompanied by phenotypic features such as aberrant electrophysiological properties and increased apoptosis [206].

In a subsequent larger-scale study involving over 100 HD-iPSC lines, astrocytes differentiated from HD-iPSCs exhibited a vacuolation phenotype, which was also observed in primary lymphocytes from HD patients [207]. Additionally, studies have demonstrated that HD-iPSC-derived NPCs, neurons, and glial cells display increased vulnerability to BDNF withdrawal, a phenomenon similar to that observed in HD animal models [208]. Among the various pathological features of HD, the loss of GABAergic MSNs in the striatum is particularly significant. Several studies have reported that GABA MSN-like neurons derived from HD-iPSCs recapitulate the key pathological features of HD, including mutated HTT aggregation, increased lysosomal and autophagosomal activity, nuclear indentations, caspase activation, and exacerbated neuronal cell death during aging [209,210,211] (Table 7).

Collectively, these findings highlight the utility of HD-iPSC-derived cell lines in modeling the diverse pathological processes and temporal progression of HD. These in vitro models provide a robust platform for investigating disease mechanisms and offer valuable opportunities for identifying and testing potential treatments for HD.

CRISPR-edited iPSC models have significantly advanced Huntington’s disease (HD) research by enabling precise genetic modifications and providing insights into disease mechanisms and potential therapies. Key modifications in the HTT gene include CAG repeat expansion, where 72 repeats showed caspase activation under stress and 97 repeats induced disease-associated phenotypes [212]. Repeat contraction using engineered Cas9 (NGN PAM) reduced pathogenic repeats [213], while allele-specific knockout through a dual gRNA approach selectively eliminated mutant HTT [214]. These genetic modifications reveal key phenotypic findings: mutant HTT expression leads to transcriptional dysregulation, impaired neurotrophic factor transport, and increased caspase activity, while genetic correction rescues neuronal viability, striatal differentiation capacity, and cellular stress responses.

### 4.4. Rare Neurodegenerative Diseases

In addition to AD, PD, and HD, iPSC-based models are also commonly used in the study of spinocerebellar ataxia (SCA) [215,216,217] and spinal muscular atrophy (SMA) [218,219,220,221], with patient-derived iPSCs exhibiting disease-specific phenotypes and vulnerabilities. For example, studies have shown that Purkinje cells in the SCA6 model exhibit susceptibility to thyroid hormone depletion-induced neurite degeneration [215]. In SMA models, survival motor neuron (SMN) protein levels in motor neurons are reduced, with degenerating neurites and delayed neural differentiation [218,219,220,221]. SMA-derived astrocytes show abnormal morphology, reduced neurotrophic factor secretion, and elevated calcium flux, which may contribute to motor neuron damage [222]. In Alexander disease models, GFAP mutations lead to protein aggregation in astrocytes, prompting these glial cells to secrete higher levels of inflammatory cytokines and other molecules, which inhibit myelination and trigger immune responses [223,224].

## 5. Conclusions

Looking forward, the continuous advancement of the iPSC technology has opened new avenues for research into neurodegenerative diseases. Studies utilizing iPSC-derived neurons, neural stem cells, glial cells, and brain organoids have provided valuable insights, particularly for disease phenotypes that manifest only in mature neurons after progressing through intermediate neural progenitor stages. Despite these advancements, the establishment of in vitro models using iPSCs remains economically challenging. This is largely attributed to the low density and limited reprogramming efficiency of fibroblasts and PBMCs, which are commonly used as source materials for iPSC generation. These limitations pose significant barriers to the large-scale application of iPSC-derived cells and organoids in drug discovery for neurodegenerative diseases. To overcome these challenges, researchers have increasingly explored direct reprogramming approaches as a promising alternative.

In summary, iPSC-based in vitro models, such as neurons and organoids, enable researchers to recapitulate neurodegenerative diseases in diverse and physiologically relevant forms, deepening our understanding of disease mechanisms. These models hold immense promise for advancing the development of targeted therapies and novel drugs, offering renewed hope for the prevention and treatment of neurodegenerative diseases.

## Figures and Tables

**Table 1:** Advantages and disadvantages of different delivery methods in the reprogramming process of iPSCs.

Methods	Advantages	Disadvantages	References
Retroviral and lentiviral vectors	Highly efficient and robust	Risk of transgene reactivation	[12]
Adenoviral vectors	Lower risk of transgene reactivation	Low reprogramming efficiency; unsuitable for clinical use	[11,13]
Sendai virus	Higher efficiency; RNA virus can be completely removed	Still involves challenges in clinical application	[11,13]
PiggyBac	Lower risk of genomic instability and mutations	Low reprogramming efficiency; limited cell sources	[14,15,16]
Minicircle vectors	Lower risk of genomic instability and mutations	Low reprogramming efficiency; limited cell sources	[14,15,16]
Episomal plasmids	No genomic integration risk; cost-effective and easy to use	Requires daily transfection; moderate efficiency	[17,18]
RNA Delivery	Lower mutagenic risk; high efficiency	Limited to specific cell types (e.g., fibroblasts, peripheral blood cells)	[19,20,21]

**Table 2:** Reprogramming factor combinations and their functional roles.

	Factors	Functions	References
Yamanaka factors	OCT3/4, SOX2, KLF4, c-MYC	OCT3/4, SOX2, and KLF4 maintain pluripotency and inhibit differentiation c-MYC enhances reprogramming efficiency and promotes cell proliferation	[23]
Alternative factor combinations	OCT3/4, SOX2, NANOG, LIN28	NANOG maintains self-renewal of stem cells LIN28 regulates RNA modification and expression	[24]
Enhancement or complementary factors	GLIS1, NR5A2, SALL4	Substitute for c-MYC or OCT3/4 to improve reprogramming efficiency and stabilize cell states	[25,26,27]
Chemical cocktails	(1) CHALP cocktail: CHIR99021, PD0325901, LIF, A-83-01, bFGF, HA-100	Enhances reprogramming efficiency	[2,28]
	(2) Alternative chemical cocktail: cyclic pifithrin-α, A-83-01, CHIR99021, thiazovivin, NaB, PD0325901	Significantly enhances reprogramming efficiency, particularly in hUCs	[2,29]
Epigenetic modulators	DNA methyltransferase inhibitors, histone deacetylase inhibitors	Regulate DNA methylation, histone acetylation, and the expression of pluripotency-associated genes to enhance reprogramming efficiency	[30,31,32]

**Table 3:** Somatic cell sources and their advantages.

Cell Type	Sources	Advantages
Fibroblasts	Skin	Readily accessible and widely used
PBMCs	Blood	Non-invasive; useful for clinical applications
Keratinocytes	Skin or hair	Non-invasive and readily accessible
MSCs	Bone marrow, adipose tissue, teeth	Abundant and frequently used in regenerative research
Renal epithelial cells	Urine	Highly convenient and non-invasive
NSCs and NPCs	Brain tissue	Inherent pluripotency; useful for neurological applications
Liver cells	Liver tissue	Expands potential applications
Stomach cells	Stomach tissue	Expands potential applications
Cord blood cells	Cord blood	Readily available from umbilical cord; useful in neonatal research

**Table 4:** Neuronal cell/organoid differentiation methods and their limitations.

Differentiated Cell/Organoid Type	Differentiation Methods	Limitations	References
NSCs/NPCs	(1) EB formation; (2) Co-culture on neural inducing feeders; (3) Dual SMAD inhibition	Requires intermediate steps; limited differentiation efficiency	[3,56,57]
Neurons (e.g., dopaminergic, GABAergic, glutamatergic)	(1) NSC/NPC stages with growth factors (BDNF, GDNF, CAMP, RA); (2) Direct conversion using transcription factors (BRN2, ASCL1, MYT1L, NGN2)	Complex protocols; variability in differentiation outcomes; phenotypic instability with direct conversion	[3,58,59,60,61,62,63,64,65,66,67,68,69,70,71,72,73,74]
Astrocytes	(1) NSC/NPC or OPC stages with CNTF, BMP, FGF2, FBS; (2) Direct induction using NFlA, NFIB, SOX9	Significant plasticity; variability across protocols; in vitro astrocytes may not fully replicate in vivo properties	[72,75,76,77,78,79,80,81,82,83,84]
Microglia	(1) Mesoderm progenitor cells with BMP4, activin A, FGF2, VEGF-A; (2) Direct induction using PU.1, IRF8	High technical complexity; multiple steps; phenotypic and functional differences from in vivo counterparts	[85,86,87,88,89,90,91,92]
Oligodendrocytes	(1) Stepwise differentiation: NPC → OPC (BMP4, FGF2, PDGF, EGF) → oligodendrocytes (T3, IGF-1); (2) Direct induction with SOX10, OLIG2, NKX6.2	Time-consuming; phenotypic instability with direct transcription factor induction; challenges in achieving purity	[93,94,95,96,97,98,99,100,101,102,103]
Brain organoids (e.g., cortical, hippocampal, midbrain, cerebellar)	Non-guided (intrinsic morphogenetic potential or guided (dual SMAD inhibition, region-specific factors such as SHH, RA, WNT inhibitors)	Lack of vascularization and heterogeneity; difficulty in precisely controlling differentiation and region specificity	[104,105,106,107,108,109,110,111,112,113,114,115,116,117,118,119,120,121,122,123,124,125]

**Table 5:** AD pathological features of different iPSC-derived models.

iPSC-Derived Model	AD’s Pathological Features	References
Neurons	– Aβ accumulation – Hyperphosphorylated Tau (p-Tau) – GSK3β activation – Abnormal electrophysiological activity – Increased oxidative stress and ROS production – ER dysfunction – Mitochondrial abnormalities – Heightened sensitivity to Aβ42 – Reduced Aβ secretion and p-Tau levels via β-/γ-secretase inhibitors	[128,131,132,133,134,135,136,137,138,139,140]
NSCs/NPCs	– Low APP and Aβ expression – Significantly impaired proliferation and self-renewal capacity – Elevated expression of neurodevelopment-related genes (e.g., MAPT, CD24, STMN2)	[131,141,142]
Microglia	– Reduced phagocytic capacity for the Aβ and Tau oligomers – Enhanced neuroinflammation	[85,143,144]
Astrocytes	– Morphological alterations – Increased Aβ42 release – Impaired clearance capacity – Dysregulated cytokine production – Calcium homeostasis imbalance – Elevated ROS levels	[145,146,147,148]
Oligodendrocytes	– Morphological defects – Impaired proliferation and functionality – Reduced neuronal support capacity – Defective myelination	[149]
3D brain organoids	– Aβ deposition – Neurofibrillary tangle formation – Enhanced Tau phosphorylation – ER abnormalities – Pathological phenotypes modifiable by pharmacological/environmental interventions	[150,151,152,153,154,155]

**Table 6:** PD pathological features of different iPSC-derived models.

iPSC-Derived Model	PD’s Pathological Features	References
DA neurons	– α-Synuclein accumulation – Mitochondrial dysfunction – Increased oxidative/ER stress sensitivity – Synaptic loss – Neuronal death – Elevated apoptosis – Impaired neuronal homeostasis – Defects in mitochondrial autophagy (e.g., PARK2/PINK1 mutants) – Elevated ROS levels – Epigenetic changes (sPD models)	[2,5,105,159,160,161,162,163,164,165,166,167,168,169,170,171,172,173,174,175,176,177,178,179,180,181,182,183,184,185,186,187,188]
Astrocytes	– α-Synuclein accumulation – Dysregulated autophagy – Abnormal mitochondrial morphology/activity – Reduced cell viability – Oxidative stress-induced DA neuron degeneration	[189,190,191]
Microglia	– Elevated α-synuclein levels – Impaired phagocytic capacity – Enhanced neuroinflammation – Disrupted DA neuron morphology/function	[191,192,193]
Neural stem cells	– Reduced differentiation efficiency – Developmental defects	[194,195]
3D brain organoids	– Increased DA neuron death – Reduced differentiation potential – Decreased dendritic complexity – Neurodevelopmental defects – Pathological phenotypes rescued by LRRK2 inhibition	[104,196,197]

**Table 7:** HD pathological features of different iPSC-derived models.

iPSC-Derived Model	HD’s Pathological Features	References
Neural progenitor/stem cells (NPCs/NSCs)	– Expression of the mutant HTT protein, CAG repeat expansion – Transcriptomic dysregulation – Aberrant electrophysiological properties – Increased apoptosis – Enhanced susceptibility to BDNF withdrawal	[206,207,208]
Forebrain neurons	– Mutant HTT aggregation – Synaptic dysfunction – Transcriptomic dysregulation	[206]
Striatal-like GABAergic neurons (MSNs)	– Mutant HTT aggregation – Increased lysosomal and autophagosomal activity – Nuclear indentations – Caspase activation – Exacerbated neuronal death during aging – Mitochondrial dysfunction (indirectly linked)	[209,210,211]
Astrocytes	– Vacuolation phenotype – Enhanced susceptibility to BDNF withdrawal – Impaired neuronal support functions	[207,208]
Glial cells (general)	– Enhanced susceptibility to BDNF withdrawal	[208]
